# Corridors best facilitate functional connectivity across a protected area network

**DOI:** 10.1038/s41598-019-47067-x

**Published:** 2019-07-26

**Authors:** Frances E. C. Stewart, Siobhan Darlington, John P. Volpe, Malcolm McAdie, Jason T. Fisher

**Affiliations:** 1 School of Environmental Studies, University of Victoria, Victoria, BC V8Z 2Y2 Canada; 2 5206 Burnham Crescent, Nanaimo, BC V9T 2H9 Canada; 3 Ecosystem Management Unit, InnoTech Alberta, Victoria, BC V8Z 7X8 Canada; 4 Present Address: Pacific Forestry Centre, Natural Resources Canada, Victoria, BC V8Z 1M5 Canada

**Keywords:** Conservation biology, Macroecology

## Abstract

Biologging data allow animal ecologists to directly measure species’ fine-scale spatiotemporal responses to environments, such as movement – critical for our understanding of biodiversity declines in the Anthropocene. Animal movement between resource patches is a behavioral expression of multiple ecological processes that affect individual fitness. Protected area (PA) networks are a tool used to conserve biodiversity by sustaining habitat patches across vast heterogeneous landscapes. However, our ability to design PA networks that conserve biodiversity relies on our accurate understanding of animal movement and functional connectivity; this understanding is rarely tested in real-world situations due to the large geographic expanse of most PA networks. Using a tractable PA network mesocosm, we employ cutting-edge biologging technology to analyze animal movement decisions in response to a highly heterogeneous landscape. We analyze these data to test, in a novel way, three common hypotheses about functional connectivity – structural corridors, least cost paths, and stepping stones. Consistently, animals moved along structurally self-similar corridors. In reference to the Aichi 2020 Biodiversity Targets, relying on species to “stepping stone” across habitat remnants may not achieve protected area network conservation objectives.

## Introduction

Researching animal ecology has always been clouded by difficulties observing free-roaming individuals. New biologging devices such as high-fix rate Global Positioning System (GPS) units quantify an animal’s space-use by providing refined observations of individual movement patterns. Movement is a behavioural expression of multiple ecological processes, the primary of which is resource selection within heterogeneous environments^1^: ecologists therefore need to fully understand this behavioural expression to implement effective biodiversity conservation tools as landscapes continue to rapidly change^2,3^. Enhanced spatial and temporal resolution in measurements of animal movement relative to landscape heterogeneity – such as how animals select, and move between, partially or wholly disjunct resource patches to meet their energetic and life-history requirements – greatly accelerates our understanding of ecological processes and animal responses to changing environments.

Increasing landscape heterogeneity through land use change – reduction and spatial fragmentation of already discontinuously distributed resources – is a global problem that affects individual animal movements, populations, and ultimately biodiversity persistence^4–7^. Animals must be able to move between disjunct resource patches to sufficiently meet life-history requirements; thus landscape-scale functional connectivity is vital for species persistence^8^. For example, through detailed and collaborative global monitoring of animal movements (i.e. Movebank.org, ICARUSinitiative.org), decreased movement in areas of high fragmentation has been observed across multiple taxa^9^. This may be in part due to increasing proportion of landscape matrix – areas of higher risk and fewer resources that animals may or may not decide to cross to access the next resource patch^10^ – within highly fragmented anthropogenic landscapes. The global area occupied by “working landscapes”– areas of high fragmentation and interspersed natural and anthropogenic features^11,12^ – far exceeds that of undeveloped protected areas^13^ so society currently relies heavily on working landscapes that contain protected areas to support biodiversity. Protected areas are often expected to anchor animal populations that use the surrounding working landscapes, and the challenges of this landscape approach to conservation is well researched^14,15^.

The IUCN’s Convention on Biological Diversity Aichi Target 11, requiring 17% of the globe’s terrestrial area to be designated as protected, is rapidly approaching (2020). To support populations and metapopulations of large vagile species (such as mammals and birds), protected areas too small for metapopulations need to be functionally connected into protected area networks (PANs)^16^. Functional connectivity is “the degree to which the landscape facilitates or impedes movement among resource patches”^17^. The majority of functional connectivity research examines the outcome of connectivity – such as gene flow, movement, or species distribution – from which functional connectivity is then inferred^18^. Without high-precision movement data, biologists struggled to reliably measure the process, rather than the outcome, of functional connectivity. Biologging helps to bridge this gap.

Here, we focus on three of the most common, non-mututally exclusive theories of connectivity; though these concepts are described under different terminologies througout the literature, we define our usage of each below.

1. The corridor framework, developed in working-landscape systems^19–21^ posits that long thin habitat patches, typically unsuitable for supporting a species on their own, will facilitate functional connectivity between populations when physically connecting structurally similar patches and habitats (i.e. resource patches)^21,22^. The classic example is wooded hedgerows connecting remnant forest patches in agricultural landscapes wherein small mammals select structurally self-similar landscape features in which to travel^20^. 2. The least-cost paths (LCP) framework is derived from ideas about light refraction^23^, and is related to electrical circuit theory^24^; defines connectivity not by physical similarity to undeveloped patches but solely by the cost a piece of landscape imparts on animal movement. Herein the landscape is a continuum of costs that correlate to the type, and density, of habitat features^25^; it does not consider habitat and matrix as binary, separate, entities. 3. The stepping stone theory is derived from Island Biogeography theory^26^. Island Biogeography, and the species-area relationship, demonstrates that species richness and turnover are outcomes of connectivity between isolated and distinct resource patches. Although controversial^27^ this theory suggests disjunct but physically proximate patches of similar resources (i.e. islands) facilitate connectivity; herein landscape matrix (i.e. oceans) and habitat (i.e. islands) are acutely distinguished, as the matrix is unusable and discrete island resource patches – represented by either polygonal landscape features, or raster cells, in a Geographic Information System – facilitate movement. In terrestrial landscapes the hostility of the matrix is relaxed but still assumed to be strongly avoided, or “stepped across”.

**Figure 1:**
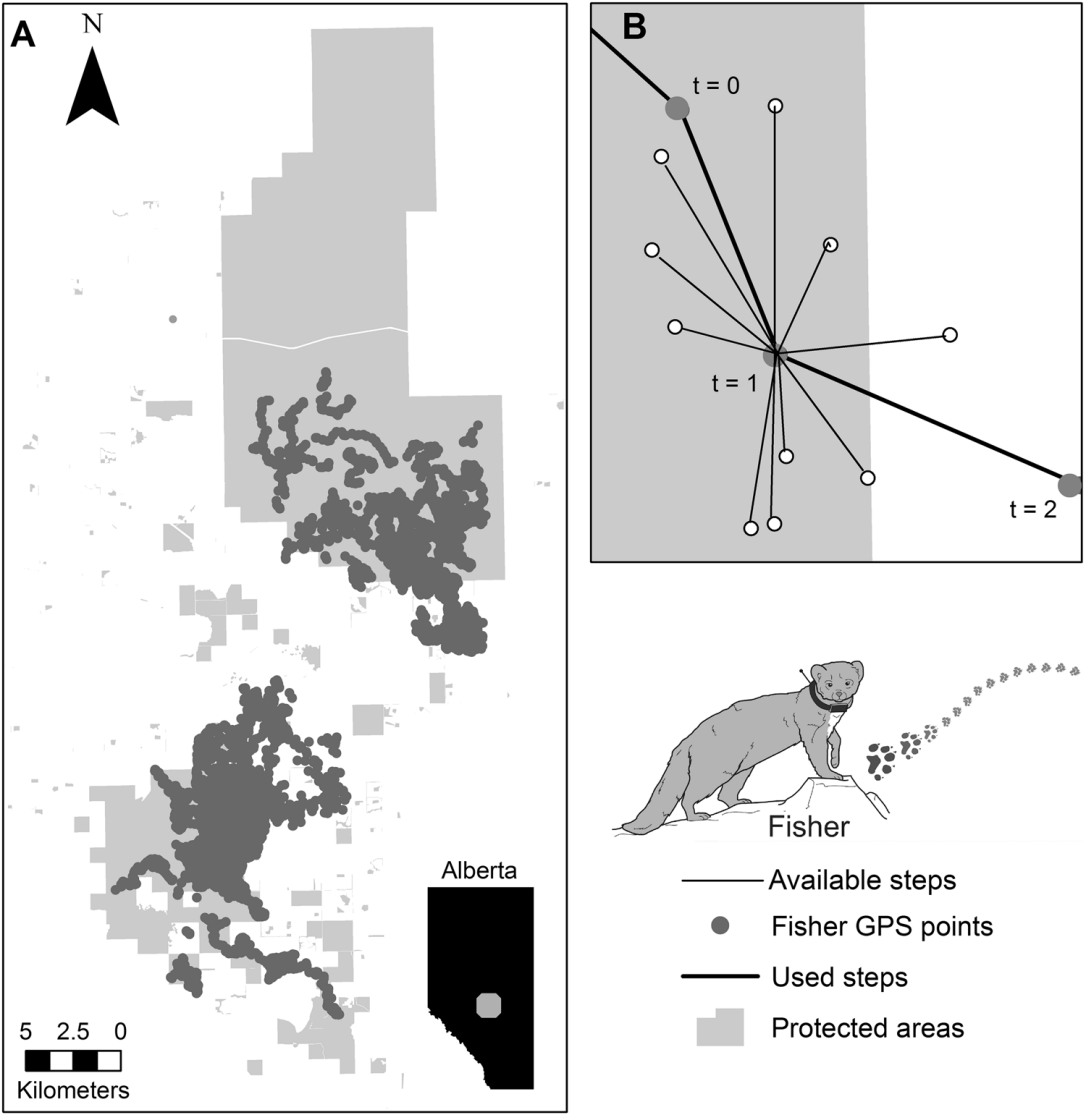
Fisher GPS telemetry locations were collected across the protected area network of the Beaver Hills Biosphere in east-central Alberta, Canada (**A**). For each used GPS step, 10 random available steps and turn angles were generated (**B**). These points were compared in a used-available, or “case-control”, design to determine the density and configuration of habitat features predicting used points.

The validity of these theories is of critical importance in landscape planning, particularly in planning new protected areas and protected area networks. Despite the increasing spread of working landscapes, and the global inititaive to increase protected areas to support biodiversity, we know very little about how well, or if, networks actually facilitate species’ movement within them (with some exceptions)^28^. We ask whether a model PA network – the newly created UNESCO Beaverhills Biosphere (Fig. 1) – facilitates functional connectivity within a working landscape. We use high-frequency biologging paired with a new movement and resource selection statistical model in a PA network landscape mesocosm^29^ to examine whether the corridor, least cost path, or stepping stone framework of connectivity best explain functional connectivity for a model species subject to extensive connectivity and biologging research^30–33^, the fisher (*Pekania pennanti)*.

**Figure 2:**
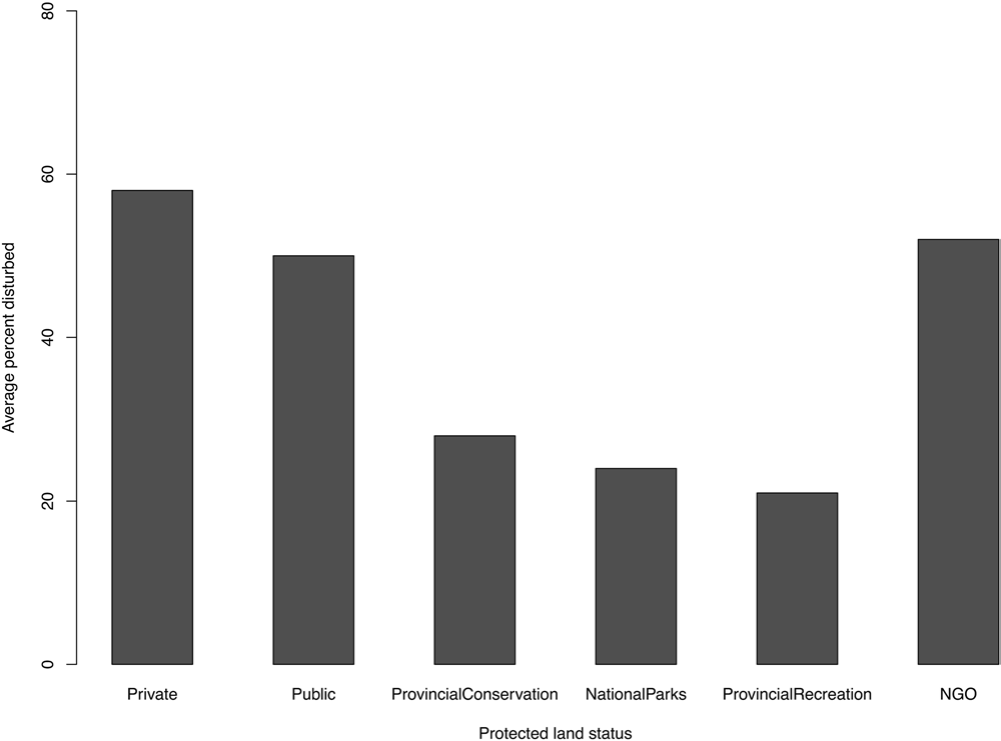
The averaged percent disturbed landscape (cultivation, development, linear, and block features) within a 500-m buffer of 64 stationary sampling sites grouped by protected area status across the Beaver Hills Biosphere, Alberta, Canada. Statuses include privately owned land, Public/County lands, Provincial Conservation lands, National Parks, Provincial Recreation lands, and Non-Government Organization (NGO) lands.

**Figure 3:**
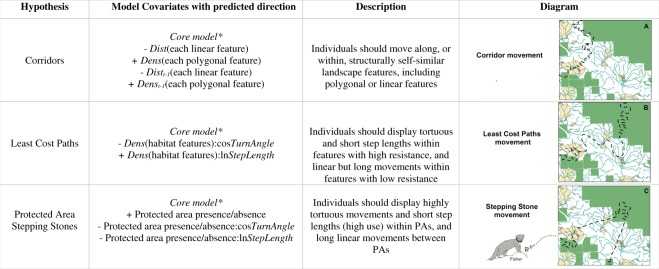
Parameters within each *clogit* model describing hypothesized frameworks for landscape connectivity across the Beaver Hills Biosphere. Distance to (*Dist*) represents fisher movement along linear features, where as density (*Dens*) represents the movement cost of a polygonal habitat feature (high density = high cost). All models involved a set of *Core model*(*) variables that we hypothesized would be generally important to fisher movement: CosTurnAngle + lnStepLength - Dist(DECID) + Dens(DECID) - Dist(CONIF) + Dens(CONIF) - Dist(MIXED) + Dens(MIXED) - Dist(WATER). Interactions are denoted by “:”.

We posed three non-mutually exclusive hypotheses and weighed evidence for each across multiple candidate sets in an information-theoretic approach. We linked hypotheses to connectivity frameworks by measuring the distance between movement steps and landscape features (measured as ‘distance to’ in meters; the proximity of animal movement to the edge of a landscape feature), and the movement cost of polygonal landscape features (measured as ‘density’ in pixels/m^2^; the cost of animal movement through landscape features of different resource densities; Fig. 3). If fishers are using protected areas as stepping stones across the working landscape matrix, we predict fisher to display highly tortuous movements and short step lengths (high use) within PAs and long linear movements between them; we expect a positive correlation between the presence of protected areas and tortuosity, and a negative correlation between the presence of protected areas and the step length (Fig. 3; Protected Area Stepping Stones). If corridors facilitate connectivity, then we predict fishers should move along structurally self-similar landscape features; we expect a positive correlation between consecutive steps so that the density of each polygonal feature, and the distance to each linear feature, is similar between steps (Fig. 3; Corridors). Finally, if fisher move along least cost pathways across the landscape, fisher should display tortuous and short step lengths within features clearly distinguished by high movement cost (high use), and linear but long movements within features with low movement costs (low use, for travel only); we expect the density of landscape features to be negatively correlated with tortuosity and positively correlated with the length of movement steps (Fig. 3; Least Cost Paths). We acknowledge that a structural corridor may indeed be a least cost pathway if it provides the path of least resistance.

## Results

Of the 14 fisher captured and collared, we obtained GPS data from 10 individuals (5 males: 5 females) comprising 17% of the estimated population^34^. These 10 collars collected 19,578 GPS fixes, over an average of 32.97 days (minimum = 4.87 days, maximum = 90.79 days) of continuous movement data per individual. Fisher step lengths over a 5-min fix interval approximated a log normal distribution with many small steps (mean = 105.47 m, s.e. = 1.85 m, min = 0.06 m, max = 2972.0 m), and turn angles were on average small and positive, indicating directional movement behaviour (mean = 0.08 rad, s.e. = 0.0001 rad).

### Corridor models containing natural features best explain movement across a heterogeneous protected area network

**Figure 4:**
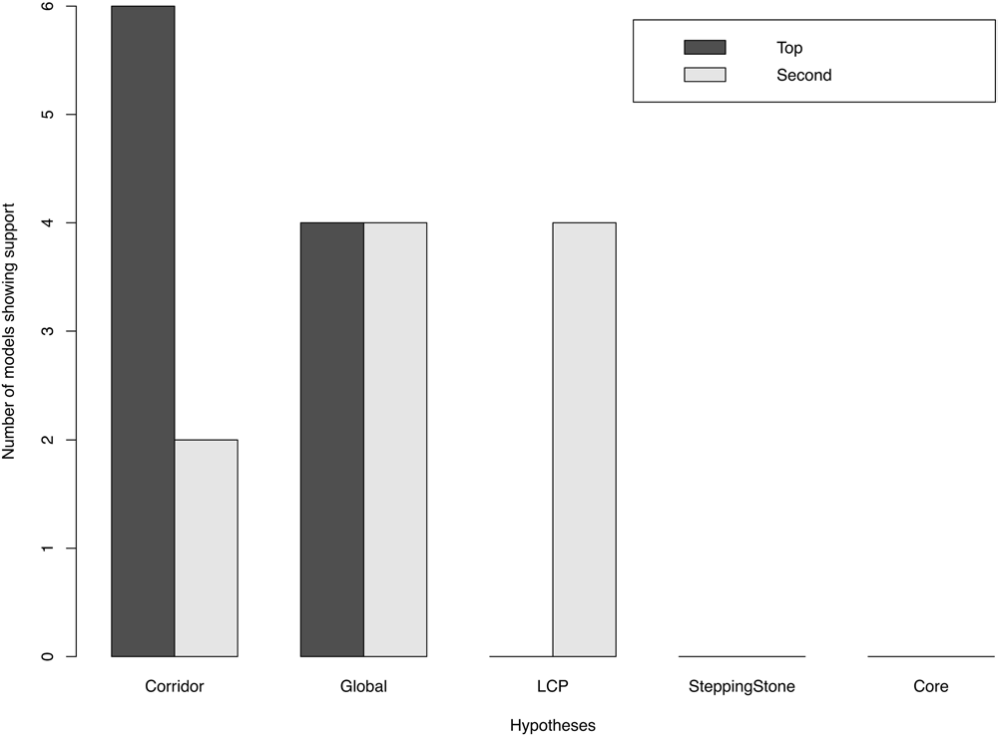
High fix-rate GPS movement telemetry data from six of 10 fisher individuals showed the highest relative support for a corridor framework of functional connectivity when compared to either least cost paths or stepping stone framework hypotheses across the heterogeneous landscape mesocosm of Alberta’s Beaver Hills Biosphere. The count of top models showing support for each hypothesis is demonstrated in black, and the count of second-best models showing support for each hypothesis is demonstrated in grey. Hypotheses include the corridor hypothesis of movement, a global model, the least cost paths hypothesis of movement, a stepping stone hypothesis of movement, and a core model representing species-specific habitat selection.

The corridor model of functional connectivity, in which animals move among structurally similar features across the landscape, best explained fisher movement across this PA network. This model received the highest AIC weight of evidence across 6 of the 10 fisher individuals (86–99%), and second highest for the 2 of the remaining 4 individuals. Four individuals showed support for the least cost paths model as the second highest AIC weight of evidence, and no individuals showed support for the stepping stone hypothesis (Fig. 4). All models generally had adequate concordance (concordance of top AIC models ranged from 0.594 to 0.647).

Among fisher individuals, polygonal natural and anthropogenic features best explained the observed variance in fisher steps. Density of protected areas, distance to nearest protected areas, and interactions between PAs and both movement step length (ln*StepLength*) and turning angle (cos*TurnAngle*) were rarely significant in top models, suggesting fishers did not distinguish the PA network from other features in the landscape (Fig. A1.1). Only two individuals’ movement was explained by PAs, but neither represented the top model for that individual. One fisher individual’s movement varied with PA density of the current (iSSA parameter slope and significance; ß = 0.20 ± 0.04, p < 0.001) and previous (ß = 0.15 ± 0.04, p < 0.001) step, suggesting that individual may have used PAs as stepping stones for components of their movement. Another fisher’s movement and habitat selection also varied positively with PA density of the previous step (ß = 0.89 ± 0.41, p = 0.04), but not of the current step: this animal left the PA network for the working landscape. Of the other eight individual fishers, three individuals had PA predictors in their top models, but these parameters were never statistically significant.

Fishers generally selected for, or remained close to, structurally similar natural features from step to step across the movement pathway (Fig. A1.1,A.1.2). This occurred despite the presence and proximity of protected areas. Together, these movement patterns suggest fishers predominantly used natural features as corridors, rather than crossing multiple landscape features with different movement costs, or using stepping stones within or between protected habitat patches.

### Individual animal responses to protected areas

If PAs conserve prime habitat patches, we predicted individual fisher to display highly tortuous and short movement steps as an indication of high residency time within PAs. However, we found variable support for this concept within individual movements: 40% of fisher displayed linear and long steps, 20% of fisher displayed linear and short steps, 20% of fisher displayed tortuous and short steps, and 20% of fisher had no variation in their steps as they only occurred in protected areas. Therefore, 40% of the data support the importance of PAs for connectivity, and 60% of the data do not. Across individual movements within PAs, step length (Pearson correlation; r = 0.003, df = 214150, p = 0.11) and turning angles (r < 0.001, df = 214150, p = 0.82) were not significantly correlated with conifer density, despite our predictions. Individuals displayed longer step lengths in PAs with dense mixedwood forests (r = 0.03, df = 214150, p < 0.001), but had no relationship with turning angle in these areas (r = < −0.001, df = 214150, p = 0.85). However, within deciduous portions of PAs, individuals displayed significantly shorter (r = −0.12, df = 214150, p < 0.001) but linear steps (r = 0.004, df = 214150, p = 0.0.2). These results, which only partially support our predictions, highlight four important points: (1) PAs may not be conserving prime habitat patches within this system, (2) unpredictability of animal responses to supposedly desirable, and therefore protected, habitats within heterogeneous environments, (3) the importance of understanding individual variation and plasticity in behavioural responses to resource acquisition under environmental variation^35^, and (4) the importance of considering matrix effects on predicted movement and behavioural variations within protected areas.

## Discussion

We unexpectedly discovered that protected areas alone had very little effect on the movement choices, and therefore landscape connectivity, of a midsized Nearctic mammal. When provided a landscape of high heterogeneity, fisher consistently used natural polygonal features as movement corridors, despite the presence of a highly heterogeneous landscape covered by a network of hundreds of protected areas of widely varying sizes. Certainly, PAs contributed to the amount of remnant native habitat on this landscape which is otherwise introgressed by agriculture, rural residency, transportation, and petroleum extraction. However, the moraine soils have made this landscape less suitable for agriculture, and hence landowners have retained native vegetation, which has proven critical for fisher movement. When planning and implementing protected area networks, the presence of multiple protected areas alone may not facilitate landscape connectivity unless structurally connected by natural, polygonal, landscape features. The ecological integrity of the rapidly developing protected areas under Aichi Target 11 may depend on the presence of natural landscape features between them; this may be of critical importance for today’s abundant working landscapes.

Measuring protected area network connectivity via outcomes – such as genetic diversity^36^ or species occurrence^37^, is of course key to understanding how landscape planning tools conserve biodiversity. However, when connectivity outcomes are shown to falter or fail, it is research on connectivity process – movement – that reveals the mechanisms behind blockages. By examining functional connectivity patterns using high-frequency biologging within a tractable mesocosm PA network, we help to understand the effect of the landscape matrix on PA network efficacy^38–40^.

The theory of Island Biogeography^26^ first provided the conceptual framework that functional connectivity of discrete habitat patches (therein, the ability of islands to receive new species, generating species richness and turnover) depends on their size and isolation from other suitable patches. In this framework, the space between habitat patches – the landscape matrix^10^ – is important, but purely inhospitable. This is true of oceanic islands, but in terrestrial studies the matrix provides a continuum of suitability^41^ that creates a spectrum of connectedness^42^. The matrix can provide varying degrees of facilitation, or impediment, for functional connectivity^38^, species richness in remnant forest patches^41^, or protected area efficacy^39^. This spectrum of connectedness in matrix facilitation is species specific^17^, but our results here also highlight that it may change for individual animals.

Animal-defined corridors are an important consideration for connectivity, as animal responses to environmental heterogeneity demonstrate individual variation^35^ and potential for plasticity^43,44^. In a similar study to ours, where the landscape matrix had a greater proportion of urban landscape, LaPoint *et al*.^31^ demonstrate fisher movement data at local scales best supports a corridor model of functional connectivity, but more importantly, that fisher-defined corridors are composed of a variety of land cover types. Our novel finding is in showing individual variation between functional connectivity frameworks, as 4 of 10 fisher iSSA models best supported a model with all possible, non-correlated, predictors (i.e. global model) rather than a model representing any specific hypothesis. This result may highlight plasticity in individual responses to environmental heterogeneity, an individual’s ability to use multiple connectivity frameworks to piece together resources (i.e. non-mutually exclusive hypotheses), or differences in PA quality among areas these fishers occupied. We demonstrate that PA network functional connectivity can be improved by incorporating individual behavioural data, rather than assuming a uniform response by individuals to structural connectivity. These ‘animal-defined’ corridors quantified in heterogeneous landscapes^45^ will help to parameterize the functional components of connectivity across seasons, and both natural (e.g. forest fires) and anthropogenic (e.g. crop rotation, development) disturbances^31,46,47^.

It is now clear that the matrix is only as valuable as its remaining natural (non-anthropogenic) habitat patches, rather than the extent and density of PAs alone, within a landscape. This is partly because land within PAs are no longer 100% natural: if suitable habitat loss, whether within or between PAs, is above 80%, matrix quality no longer buffers extinction thresholds^40^. Andren^48^ demonstrated a similar finding across birds and mammals; he attributes this finding to the fact that above 70% of habitat loss, percolation theory predicts that the effects of habitat loss and habitat fragmentation will compound. Corridors are meant to overcome this “percolation effect” by forcing connections between habitat patches and thus preventing the compounding effects of habitat loss and habitat fragmentation. Here, in our empirical test of these concepts, prioritizing natural features between PAs best facilitates functional connectivity within PA networks by providing corridors for fisher movement. We contend this result is very likely similar for other forest-dependent species that comprise the bulk of biodiversity on this^49,50^, and other, Nearctic forested landscapes.

## Conclusions

There is substantial monetary and political capital investment in PA protection^13^. However, the investment typically ends at the PA border, and mechanics of biodiversity conservation in the matrix is left purely to hope. Here, we test ecological theory to show that conservation tools cannot rely on either proximity or hope – we need to better understand animal responses to environmental heterogeneity, and we need a planned and protected matrix designed from correct theoretical underpinnings to provide effective biodiversity conservation across PA networks.

The current state of biologging science allows for data collection that solidifies the underpinnings of connectivity conservation. Combining these high-resolution data (e.g. GPS telemetry on Movebank.org), with detailed GIS data within an iSSA framework, allows for increased precision and sophistication than ever before for testing long-standing hypotheses with ecological data. Our PA network mesocosm analysis suggests that natural habitat within landscape matrices are just as important as natural habitat within protected areas: consideration of natural features within the matrix should receive greater management consideration. In addition to creating new protected areas under the Aichi Target 11^16^, focusing on maintaining or restoring natural landscape features within the matrix of existing PA networks, or creating PA networks within existing landscapes of high natural features, will greatly aid conservation objectives. Using biologging, these objectives can now be regularly monitored and adapted as necessary within an adaptive management framework^51^, elucidating important patterns that challenge our conceptual understanding of animal ecology and conservation science. We show that increasing the extent of the global protected area network is not a stand-alone solution to connecting protected areas; the conservation of natural landscape features between PAs is the mortar that binds them together.

## Materials and Methods

### Data collection across the mesocosm

The Beaver Hills Biosphere (BHB) covers approximately 1,596 km^2^ of glacial moraine in east-central Alberta, Canada (53.381167°N, 113.062976°W; Fig. 1). This heterogeneous landscape is composed of natural, anthropogenic, and protected area (PA) habitats (Table 1). Natural habitats can be either inside or outside of PAs and consists primarily of native aspen parkland (*Populus tremuloides* and *P. balsamifera*), and interspersed small waterbodies, meadows, and patches of white spruce (*Picea glauca*). Seven hundred and sixty-three PAs of varying size (mean ± SE = 78.4 ± 29.0 km^2^), status (from local conservation easements managed by non-government organizations to provincial and national parks), and isolation (measured as the distance between protected areas; mean ± SE = 0.95 ± 0.004 km) conserve these native features across the BHB – but these PAs also have a degree of anthropogenic development within them, including grazing areas, extensive recreation, and roads (Fig. 2). The rest of the landscape is composed of extensive resource extraction in the form of oil and gas, agriculture, forestry, and rural-residential development. The resulting matrix surrounding the BHB’s protected areas is a mosaic of private lands, roads, and agriculture that separate the BHB from tracts of contiguous forest in other parts of the province.

**Table 1:** Distance to (*Dist*), and density around (*Dens*), the end of both used and available fisher steps were quantified across 15 landscape features within the Beaver Hills Biosphere.

Category	Landscape feature	Feature type	Description
Natural features	Bare	Polygonal	Distance to, and density, of exposed land
	Deciduous forests	Polygonal	Distance to, and density, of deciduous forest; native natural forest stands of primarily aspen or balsam poplar
	Coniferous forests	Polygonal	Distance to, and density, of coniferous forest; native natural forest stands of primarily white or black spruce
	Mixed forests	Polygonal	Distance to, and density, of mixed forests; native natural forest stands of mixed deciduous and coniferous species
	Wetlands	Polygonal	Distance to, and density, of water bodies; wetlands and ephemeral lakes
	Grasslands	Polygonal	Distance to, and density, of grassland; native natural grass cover
	Lakes	Polygonal	Distance to, and density, of water bodies; lakes
	Shrubs	Polygonal	Distance to, and density, of shrub-land; native natural shrub cover
	Streams	Linear	Distance to, and density, of water bodies; streams and small rivers
Anthropogenic features	Development	Polygonal	Distance to, and density, of built-up land (e.g. residential, municipal, or commercial)
	Crops	Polygonal	Distance to, and density, of annual and perennial crops
	Forage	Polygonal	Distance to, and density, of pastures and forages
	Rail lines	Linear	Distance to, and density, of rail transport lines
	Roads	Linear	Distance to, and density, of hard roads, vegetated roads, and trails
Protected areas	Protected areas	Polygonal	Distance to, and density, of parks and protected areas; protected area of any status (e.g. public lands, provincial parks, provincial recreation areas, national parks, conservation areas, and NGO sites)

From November 2015 through February 2016 we used covered cage traps (Tomahawk 109, Tomahawk, WI) to live-capture 10 fisher^50^. We used a combination of ketamine (concentration = 100 mg/ml, dose = 12 mg/kg) and midazolam (concentration = 5 mg/ml, dose = 0.3 mg/kg) to sedate individuals; we monitored vital rates and fitted individuals with GPS tracking collars (E-obs Collar 1 A; Grünwald, Germany). Collars contained a GPS microchip, ultra-high frequency transmitter for telemetry and data download, and tri-axial accelerometer; the GPS was programmed to take a GPS-fix every 5 minutes if the individual was moving greater than 10 cm/s. Spatial capture-recapture modeling of these data estimate the BHB fisher population to be at most 58 individuals (3.91 fishers/100 km^2^)^34^. We therefore obtained GPS telemetry data from at least 17% of the contemporary population. All research was approved by the InnoTech Alberta Animal Care Committee (2070M-A02/048/15-P01), and all research methods were performed in accordance with Canadian Council on Animal Care.

### Integrated step selection analysis

Functional connectivity can be measured as movement of animals in relation to landscape structure. The used movement path of individual animals can be obtained from GPS-fixes collected from GPS-telemetry collars. For each used GPS-fix within this study, we generated 10 random available steps and turn angles, and compared in a used-available, or “case-control”, design^52^ (Fig. 1). These observed steps and turn angles were assigned a “1”, whereas available (i.e. generated in GME) steps and turn angles a “0” and together represent the binomial response variable in our conditional logistic regressions^52^.

Step lengths, which are defined as straight-line distances between successive GPS-fixes, measure the speed of an animal (i.e. m/5 min) and can be used as an estimate of animal residency time within habitat features: shorter steps indicate longer residency time^53,54^. Using the *movement.ssf* function in GME (www.spatialeclogy.com/gme/), available fisher step lengths were sampled from a log normal distribution parameterized on used step lengths for each individual (distribution shape varied between 3.31–4.45, distribution scale varied between 1.99–1.40). Step lengths were ln-transformed (ln*StepLength*) *sensu* Avgar *et al*.^52,55^, and are an estimator of the selection-free speed of animal movement. Available turn angles were sampled from a uniform distribution between –π and π radians^52^ and are defined as the angular deviation between two headings; these values were cosine-transformed (cos*TurnAngle*), which transitions a circular measure (radians) into a linear measure between −1 and 1^55,56^; values approaching 1 represent linear movement^57^. Therefore, steps without a proceeding step (i.e. the first step collected for each individual) were removed from the analysis. We conducted an analysis examining habitat selection and movement for each of 10 individuals to quantify support for each connectivity framework across the BHB.

#### Landscape features as model covariates

To test the effect of landscape features on step length, we used ArcGIS v10.3 (ESRI, Redlands, CA, USA) Geographic Information System to quantify landscape heterogeneity. We used the LandSat digital map inventory from the Beaver Hills Biosphere (Land Management Framework 2015) to quantify the distance of the end points of fisher steps (m) to each landscape feature, as well as the density of landscape features, across 15 categories representing natural, anthropogenic, and protected areas (PAs); bare landscape, crops, deciduous forests, mixed forests, coniferous forests, wetlands, development, forage, grasslands, lakes, shrubs, protected areas, rail lines, roads, streams, and PAs (Table 1). Landscape features, whether polygonal or linear, were converted to a raster to calculate density; they were measured as the density of a buffer around the end point of each step, wherein the buffer radius was determined by the mean fisher step length (106 m) and measured as the number of raster pixels/m^2^. We scaled these measures to allow comparison of coefficients within regression models as a measure of explained variance; these scaled distances, and density, measures comprise the predictor variables in our conditional logistic regressions.

Our final data set comprised 214,148 used and available steps across 10 fisher individuals, including (1) used/available status, step length, turn angle, fisher ID, UTM coordinates, and strata of available steps; and (2) the relationship of each step to landscape predictor variables – either the distance to, and/or density, of 15 landscape features (Table 1). Distance to was truncated to 1000 m, as we did not suspect fisher to respond to landscape features that were any further way.

### Statistical analyses

We created statistical models to test the three hypotheses of landscape connectivity derived from current analysis methods: corridors, least cost paths, and stepping stones (Fig. 3). We developed a ‘core model’ of assumed habitat features explaining variation of fisher movement in a non-human dominated landscape and included this model within each of our connectivity hypotheses (Fig. 3). ln*StepLength* (m) represents the linear displacement between consecutive steps – a proxy for animal speed as the time between steps is constant (m/5-minutes) – whereas the movement directionality, or tortuosity, is described by the cosine of the turning angle^58^. Including ln*StepLength* and cos*TurnAngle* as model predictors within a clogit regression extends the step selection function framework^53^ into an integrated Step Selection Analysis (iSSA), accounting for animal movement speed and directionality within selected habitat features^52^.

From previous research we expect tortuosity (i.e. turn angle) and speed (i.e. step length) to affect fisher movement and fisher to select areas of deciduous forest, coniferous forest, and mixed forest, while remaining proximate to water bodies^31,32,59,60^. These are what we define as ‘high’ movement cost habitats for fisher; landscape features that receive a high proportion of residency time. We therefore included the cos*TurnAngle*, ln*StepLength*, distance to wetlands, deciduous, coniferous, and mixed forests, as well as density of deciduous, coniferous, and mixed forests within our core model (Fig. 3).

We competed five conditional logistic regression models (Fig. 3) in an Information Theoretic approach using Akaike Information Criterion values^61^ (AIC; Fig. 3) for each fisher individual. In a similar approach to Prokopenko *et al*.^55^ we used the *clogit* function in the *Survival* package^62^ in R (v3.2.2)^63^, to perform conditional logistic regression models; the response variable was steps observed (0/1), and each strata was assigned to paired used:available steps (1/0)^55^. We thoroughly explored our data^64^ ensuring all *clogit* model assumptions were met. All statistical analyses were conducted in R^63^, and results are presented as mean ± SE unless otherwise specified.

## Supplementary information

Appendix 1. Direction of selection (ß) across individual-specific fisher iSSA parameters

## Data Availability

The datasets generated and analyzed during the current study are available from the corresponding author upon reasonable request.
