# Supplementary material for: Corridors best facilitate functional connectivity across a protected area network
Source: Sci Rep. 2019 Jul 26;9:10852. doi: 10.1038/s41598-019-47067-x (PMC6659697; doi:10.1038/s41598-019-47067-x)
Supplement: Supplementary file 1 — Appendix 1. Direction of selection (ß) across individual-specific fisher iSSA parameters [file 41598_2019_47067_MOESM1_ESM.pdf]

Corridors best facilitate functional connectivity across a protected area network

Frances E.C. Stewart[1*], Siobhan Darlington[1], John P. Volpe[1], Malcolm McAdie[2], and Jason T. Fisher[1,3]

[1]*School of Environmental Studies, University of Victoria, Victoria, BC, Canada, V8Z 2Y2*
[2]*5206 Burnham Crescent, Nanaimo, BC, Canada, V9T 2H9*
[3]*Ecosystem Management Unit, InnoTech Alberta, Victoria, BC, Canada, V8Z 7X8*

Appendix 1. Direction of selection (ß) across individual-specific fisher iSSA parameters

Figure A1.1. Box plots of beta coefficients for the Corridor model iSSA parameters across all individual fisher that best supported the Corridor hypothesis (N = 6) across the Beaver Hills Biosphere of Alberta, Canada. Parameters include, fisher speed and tortuosity (white), Natural landscape disturbance parameters (green), anthropogenic landscape disturbance parameters (brown), and protected area parameters (blue). All parameters were measured as both 'density' (pixels/m^2), and 'distance to' (m), consecutive steps for each natural, anthropogenic, and protected area parameter (Density, Density_t1 and Distance, Distance_t1).

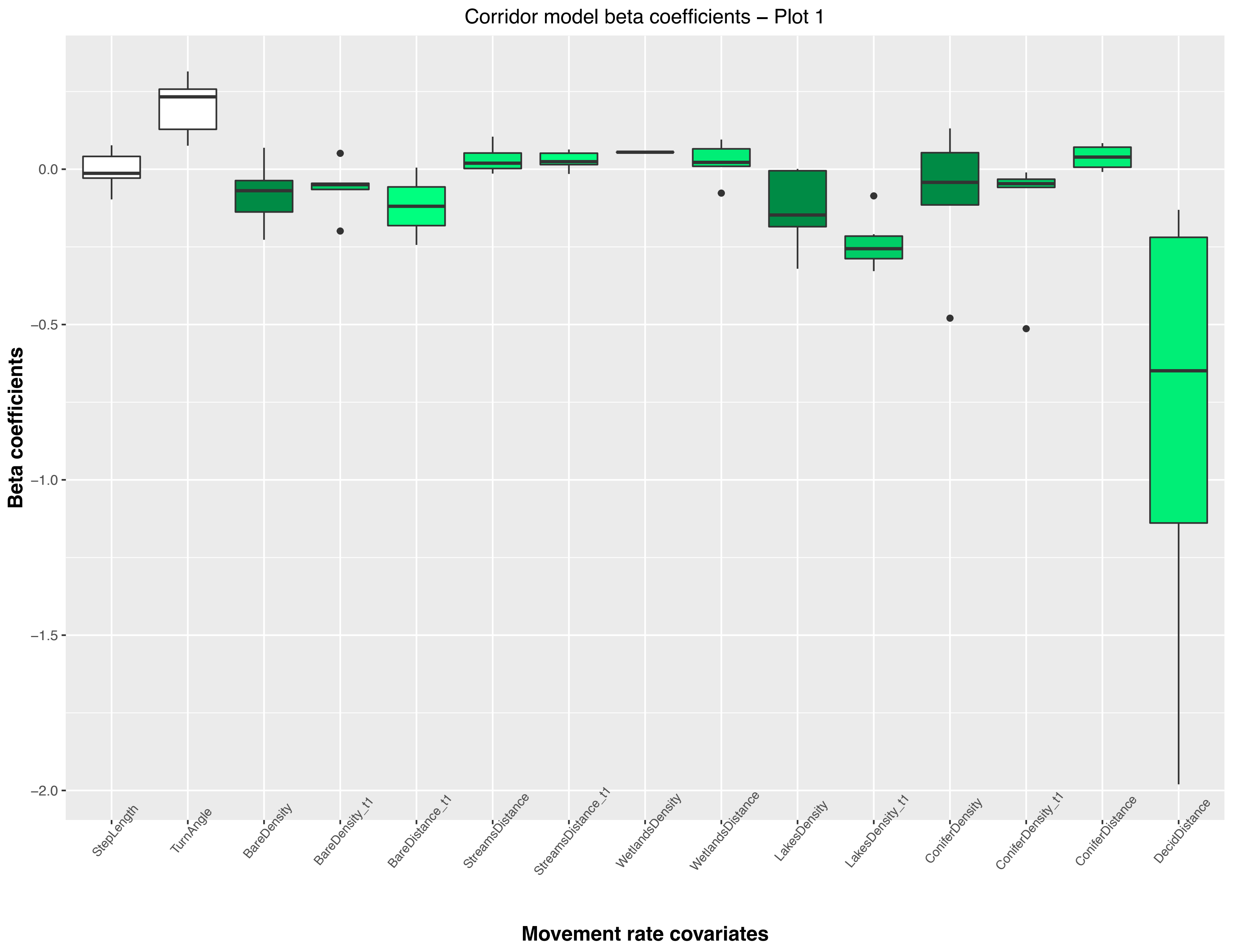

Corridor model beta coefficients – Plot 1
Beta coefficients
0.0
−0.5
−1.0
−1.5
−2.0
StepLength
TurnAngle
BareDensity
BareDensity_t1
BareDistance_t1
StreamsDistance
StreamsDistance_t1
WetlandsDensity
WetlandsDistance
LakesDensity
LakesDensity_t1
ConiferDensity
ConiferDensity_t1
ConiferDistance
DecidDistance
Movement rate covariates

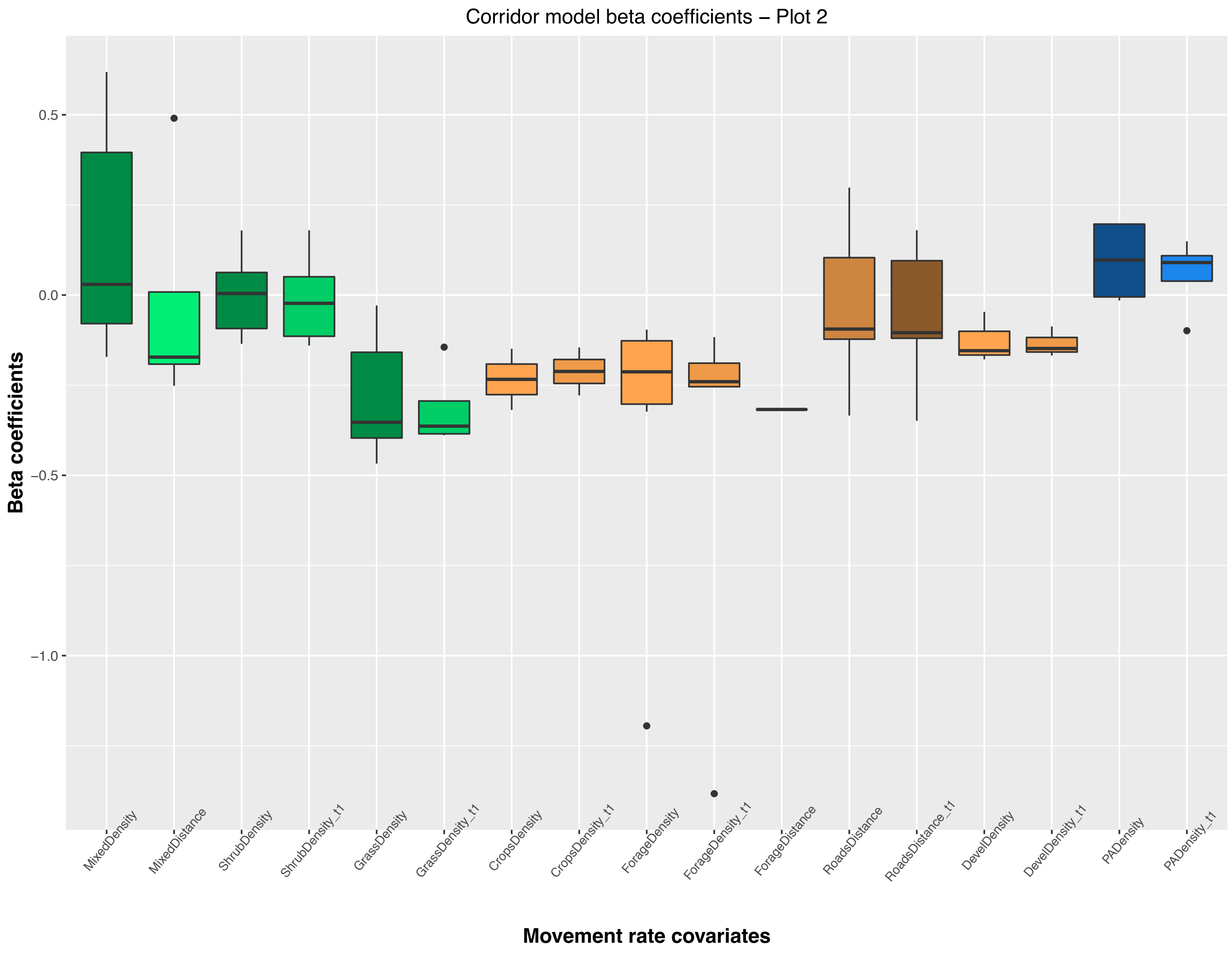
Corridor model beta coefficients – Plot 2
Beta coefficients
0.5
0.0
−0.5
−1.0
MixedDensity
MixedDistance
ShrubDensity
ShrubDensity_t1
GrassDensity
GrassDensity_t1
CropsDensity
CropsDensity_t1
ForageDensity
ForageDensity_t1
ForageDistance
RoadsDistance
RoadsDistance_t1
DevelDensity
DevelDensity_t1
PADensity
PADensity_t1
Movement rate covariates

Figure A1.2. Box plots of beta coefficients for the Least Cost Paths (LCP) model iSSA parameters across all individual fisher that best supported the LCP hypothesis (N = 6) across the Beaver Hills Biosphere of Alberta, Canada. Parameters include, fisher speed and tortuosity (white), Natural landscape disturbance parameters (green), anthropogenic landscape disturbance parameters (brown), and protected area parameters (blue). All parameters were measured as both ‘density’ (pixels/m^2), and ‘distance to’ (m), movement steps for each parameter; Plot 1. Plots 2 and 3 investigate the interaction between parameters and fisher StepLength (Plot2), and TurnAngle (Plot3).

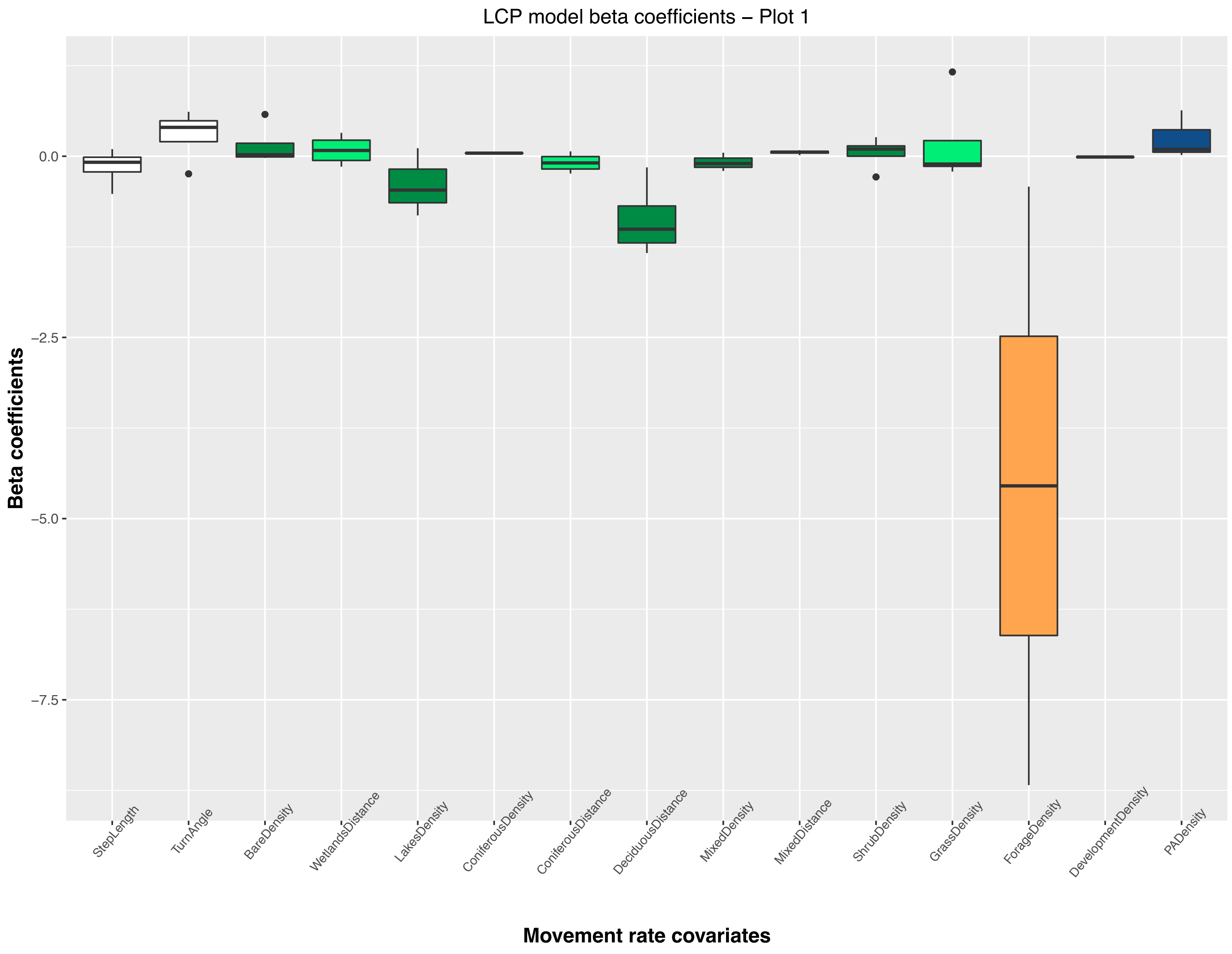

LCP model beta coefficients – Plot 1
Beta coefficients
0.0
−2.5
−5.0
−7.5
StepLength
TurnAngle
BareDensity
WetlandsDistance
LakesDensity
ConiferousDensity
ConiferousDistance
DeciduousDistance
MixedDensity
MixedDistance
ShrubDensity
GrassDensity
ForageDensity
DevelopmentDensity
PADensity
Movement rate covariates

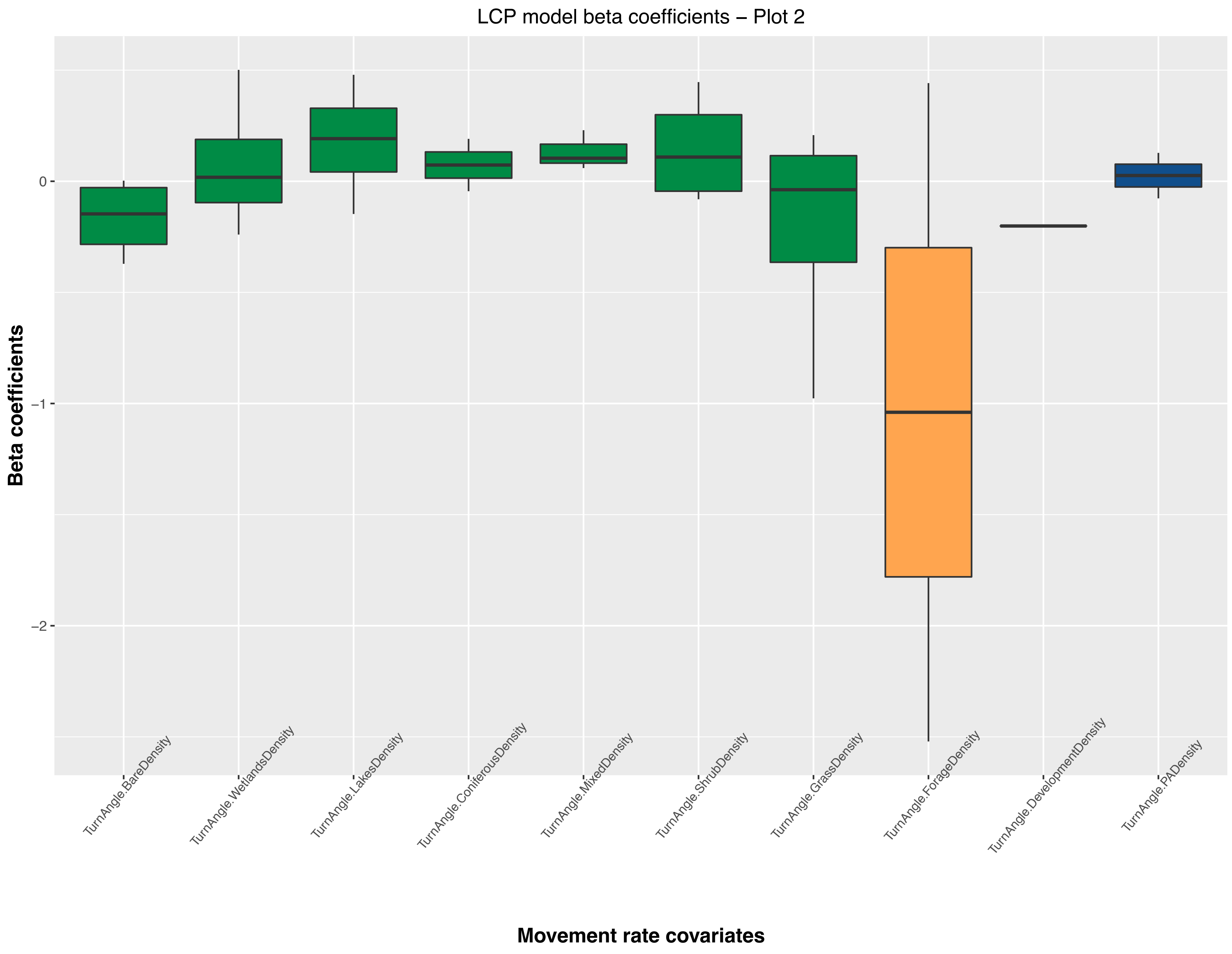
LCP model beta coefficients – Plot 2
Beta coefficients
0
−1
−2
TurnAngle.BareDensity
TurnAngle.WetlandsDensity
TurnAngle.LakesDensity
TurnAngle.ConiferousDensity
TurnAngle.MixedDensity
TurnAngle.ShrubDensity
TurnAngle.GrassDensity
TurnAngle.ForageDensity
TurnAngle.DevelopmentDensity
TurnAngle.PADensity
Movement rate covariates

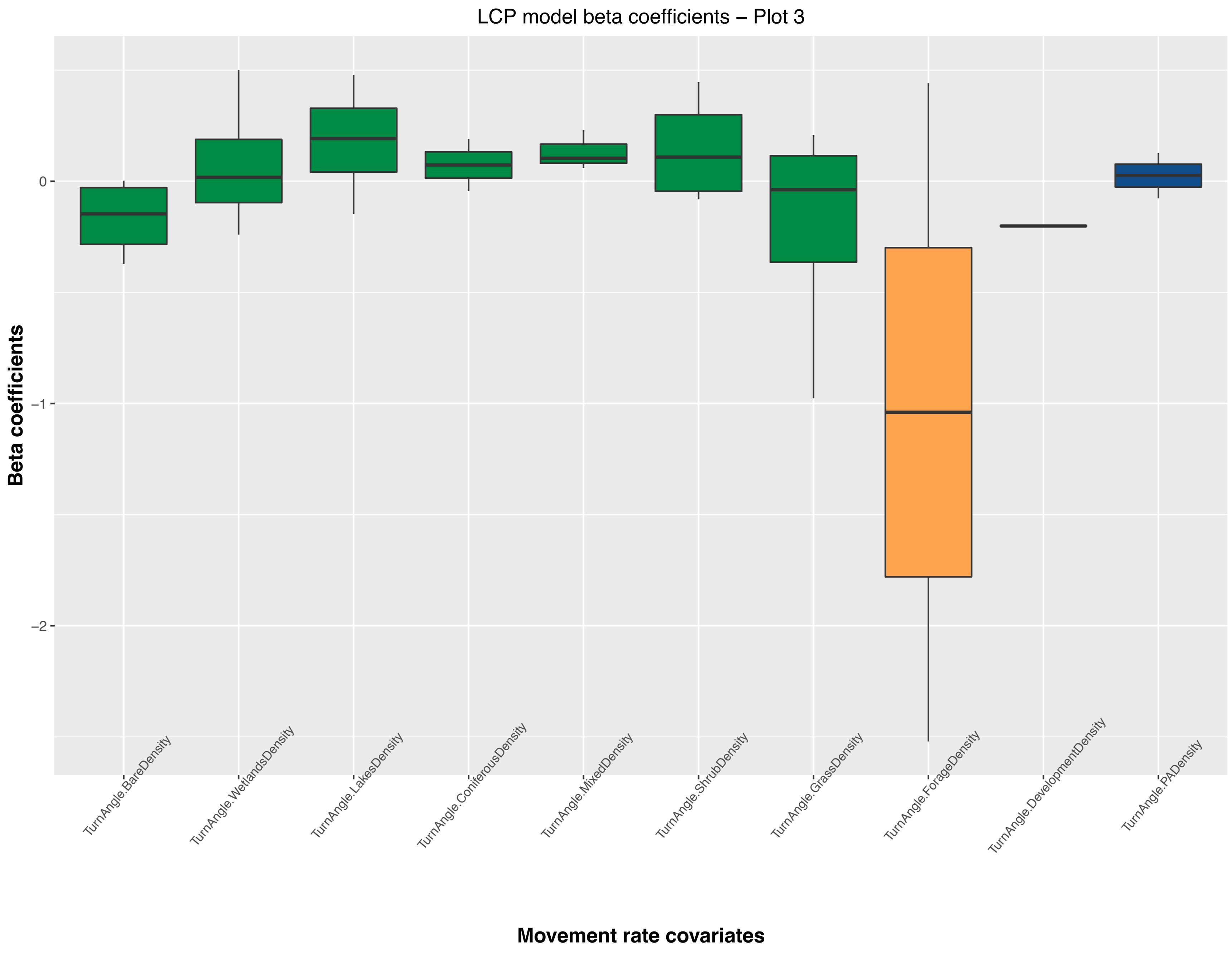
LCP model beta coefficients – Plot 3
Beta coefficients
0
−1
−2
TurnAngle.BareDensity
TurnAngle.WetlandsDensity
TurnAngle.LakesDensity
TurnAngle.ConiferousDensity
TurnAngle.MixedDensity
TurnAngle.ShrubDensity
TurnAngle.GrassDensity
TurnAngle.ForageDensity
TurnAngle.DevelopmentDensity
TurnAngle.PADensity
Movement rate covariates
